# Genome-wide identification of Hsp70/110 genes in rainbow trout and their regulated expression in response to heat stress

**DOI:** 10.7717/peerj.10022

**Published:** 2020-10-23

**Authors:** Fang Ma, Lintong Luo

**Affiliations:** College of Biological Engineering and Technology, Tianshui Normal University, Tianshui, Qinzhou District, China

**Keywords:** Rainbow trout, Gene expression pattern, Heat stress, Hsp70/110 gene

## Abstract

Heat shock proteins (Hsps) play an important role in many biological processes. However, as a typical cold water fish, the systematic identification of Hsp70/110 gene family of rainbow trout (*Oncorhynchus mykiss*) has not been reported, and the role of Hsp70/110 gene in the evolution of rainbow trout has not been described systematically. In this study, bioinformatics methods were used to analyze the Hsp70/110 gene family of rainbow trout. A total of 16 hsp70/110 genes were identified and classified into ten subgroups. The 16 Hsp70/110 genes were all distributed on chromosomes 2, 4, 8 and 13. The molecular weight is ranged from 78.93 to 91.39 kD. Gene structure and motif composition are relatively conserved in each subgroup. According to RNA-seq analysis of rainbow trout liver and head kidney, a total of four out of 16 genes were significantly upregulated in liver under heat stress, and a total of seven out of 16 genes were significantly upregulated in head kidney. RT-qPCR was carried out on these gene, and the result were consistent with those of RNA-seq. The significantly regulated expressions of Hsp70/110 genes under heat stress indicats that Hsp70/110 genes are involved in heat stress response in rainbow trout. This systematic analysis provided valuable information about the diverse roles of Hsp70/110 in the evolution of teleost, which will contribute to the functional characterization of Hsp70/110 genes in further research.

## Introduction

Heat Shock Proteins (HSPs) are a highly conserved family of proteins that maintain cell homeostasis and protect cellular function when the body is subjected to chronic or acute stress (Craig & Gross, 1991). They have been found in large quantities in eukaryotes and prokaryotes, and their expression levels are very low under normal physiological conditions (Craig & Gross, 1991). As a molecular chaperone, it is involved in physiological activities such as protein folding, assembly of protein polypeptide chain, refolding of denatured protein, immune recognition and apoptosis, and protects the body through repairing denatured protein (Sreedharan & Van, 2016). The synthesis of HSPs increased rapidly under the environmental stress including ultraviolet irradiation, heat shock, heavy metals, and pathological conditions (Lindquist & Craig, 1988).

According to the molecular weight, the Hsps are divided into Hsp110, Hsp90, Hsp70, Hsp60, Hsp40, Hsp10, and small Hsps (Li & Srivastava, 2004). Among them, the most widely studied heat shock protein is about 70 kDa, heat shock protein 70s (Hsp70s). A large amount of study has reported that the expression pattern of Hsp70 gene can be induced by various stress factors (Arai et al., 1995; Liu et al., 2014). They play an important role, not only in protein folding, membrane, translocated, misfolded, protein degradation and other cell-protecting processes, but also responses to stress, bacterial infection, parasitism and inflammatory (Qin et al., 2013). Members of the Hsp70 gene family has been systematically described in a number of species, such as Humans, birds, amphibians, zebrafish, catfish, and medaka (Song et al., 2016). In addition, Hsp110, have a high homology with Hsp70 members except for a non-functional insertion in SBD and C-terminal extension (Shaner et al., 2004; Liu & Hendrickson, 2007). Hsp110 members are considered to be nucleotide exchange factors in the Hsp70 family (Kampinga et al., 2009), and Hspa4 and Hspa4L members of Hsp110 are even named hspa members in the Entrez Gene database (Kampinga et al., 2009). Together with hsp70, hsp110 participates in the biological processes of protein folding and renaturation, degradation and translocation (Xu et al., 2012). Therefore, hsp110s have been studied together with hsp70s as an Hsp70/110 family (Kampinga et al., 2009). Deane & Woo (2011) analyzed the Hsp genes of 91 species of fishes and found that two-thirds of them belonged to HSP70 families. Two different cDNA subtypes of Hsp70 were isolated and identified from zebrafish and rainbow trout. Compared with other vertebrates such as humans, the amino acid sequence of fish Hsp70 is highly homologous with that of humans (Deane & Woo, 2011).

Similar to mammals, Hsp70 in fish is produced by a variety of physiological and environmental stresses (Ming et al., 2010). Teleost underwent teleost-specific whole genome duplication (TSGD or 3RWGD) events during evolution (Meyer & Van de Peer, 2005). Therefore, there are more Hsp70/110 genes in teleost than in mammals and other vertebrates, and most of the Hsp70/110 genes are described in teleost. Data were collected as previously described in Ma et al. (2019), rainbow trout, as a typical cold-water fish, which has poor tolerance to high water temperature, and the growth, behavior and reproduction of rainbow trout are affected by temperature changes. The optimum growth temperature is 12−18 °C (Kaya & Calvin, 1978), and the survival will be threatened when the water temperature exceeds 25 °C (Matthews & Berg, 1997). The liver and head kidney are crucial metabolic organs in fish, in which gene transcription is known to be responsive to temperature changes (Hori et al., 2010; Li et al., 2017). Thus, liver and head kidney were selected as model organs in the present study.

Although there have been many studies on the molecular biology of fish Hsp70/110 (Lund & Tufts, 2003; Ojima, Yamashita & Watabe, 2005), studies on the synthesis mechanism of Hsp70/110 in stress and the total number of genes encoding Hsp70/110 family members in rainbow trout genome and their chromosome determination are still limited. In recent years, the genome resources of rainbow trout have been well developed. The transcription data generated by RNA-seq and the results of genome sequencing (http://www.genoscope.cns.fr/trout/data/) make it possible to systematically analyze the Hsp70/110 family genes in rainbow trout genome. Therefore, in this study, we identified the Hsp70/110 gene family and evaluated the expression of Hsp70/110 under heat stress to study the effect of high temperature on rainbow trout and provide theoretical basis for the study of heat stress resistance of rainbow trout.

## Materials and Methods

### Identification of the Hsp70/110 gene family in rainbow trout (*Oncorhynchus mykiss*)

To identify the Hsp70/110 genes, First, the coding sequence (CDS)and Full-length of Hsp70/110 of Atlantic salmon (*Salmo salar*), zebrafish (*Danio rerio*), medaka (*Oryzias latipes*), tilapia (*Oreochromis niloticus*), lizard (*Anolis carolinensis*), chicken (*Gallus gallus*), mouse (*Mus musculus*) and human (*Homo sapiens*) (Table S1) were downloaded from NCBI (http://www.ncbi.nlm.nih.gov) and Ensembl (http://www.ensembl.org) databases as query sequences to search against whole genome of rainbow trout (*Oncorhynchus mykiss*) and RNA-seq results of liver and head kidney from our research group. Second, a hidden Markov model (HMM) profile of the Hsp70/110s were employed to query the rainbow trout dataset using HMMER software (Finn, Clements & Eddy, 2011; Eddy, 2011). Download the HMM profile from the Pfam protein family database (http://pfam.xfam.org/). The e-value was set at an intermediately stringent level of e^−10^ for collecting as many as potential Hsp70/110 and then the information of Hsp70/110 genome members of rainbow trout (*Oncorhynchus mykiss*) was obtained. The online program (SMART) (http://smart.embl-heidelberg.de/) and Pfam (https://www.ebi.ac.uk/Tools/hmmer/) were used to confirm the presence of the structural domains of the candidate proteins, and DNAMAN alignment screening was carried out for all the predicted Hsp70/110 proteins sequences to delete redundant sequences. The amino acid identity and similarity of Hsp70/110 from rainbow trout were generated by EMBOSS Water software (https://www.ebi.ac.uk/Tools/psa/emboss_water/).

### Physicochemical properties analysis of the Hsp70/110 genes

The Prot Param (http://web.expasy.org/protparam/) was used to analyze the Protein length, molecular weight, isoelectric point (pI) and total average hydrophilicity (GRAVY) of rainbow trout (*Oncorhynchus mykiss*) Hsp70/110 family proteins.

### Phylogenetic analysis of the Hsp70/110 genes

The protein sequences of Hsp70/110 from rainbow trout (*Oncorhynchus mykiss*) and other vertebrate organisms were used to construct a phylogenetic tree. Accession numbers for all sequences of other vertebrate organisms are provided in Table S1. Clustal W2 program (Larkin et al., 2007) with default parameters was used to alignment multiple protein sequences before the construction of a phylogenetic tree. The phylogenetic tree for the Hsp70/110 proteins was constructed using neighbor-joining (NJ) method of MEGA7.0 program (Kumar, Stecher & Tamura, 2016) with p-distance and bootstrap value was set to 1,000 replicates.

### Motif recognition and gene Exon-Intron structure analysis

MEME (http://meme-suite.org/tools/meme) was used to predict the conservative element (motif) of rainbow trout (*Oncorhynchus mykiss*) Hsp70/110 gene family. The parameters chosen were as follows: maximum length of the conserved motif, 50; minimum length, 6; largest number, 10. Genetic exon-intron structure of Hsp70/110 genes was conducted using GSDS (http://gsds.gao-lab.org/) by comparing CDSs and their corresponding genomic sequences.

### Subcellular localization and Three-Dimensional Structure analysis of Hsp70/110 Proteins

Subcellular localization was performed by WoLF PSORT (http://www.genscript.com/wolf-psort.html). The amino acid sequences of the rainbow trout Hsp70/110 were submitted to Phyre 2 (http://www.sbg.bio.ic.ac.uk/phyre2/html/page.cgi?id=index) to generate 3D structure models and we chose the best model with the highest confidence. The predicted model was further analyzed with Chimera 1.2 (Pettersen et al., 2004), showing the proportion of alpha helix, beta turn and random coil.

### Fish conditions and sampling

In this study, data were collected as previously described in Ma et al. (2019). In brief, 120 fish with the same genetic background and average body weight of (400+10.5) g were obtained from a trout-cage aquafarm in Liujiaxia Reservoir, Gansu, north-western China. Fish were maintained at a temperature of 18 °C in a 3000 L aerated water tank for 7 days and were randomly divided into six groups and adapted for another 7 days at 18 °C. At each stage of culturing, fish were exposed to a constant 12 h light and 12 h dark photoperiod and fed twice daily. Fish were then subjected to thermal stress by continually increasing the temperature at a rate of 1 °C/24 h, from 18 °C to 24 °C, using a warm heater and heating rods. After euthanasiaingi fish with a lethal dose of MS-222, liver and head kidney samples were harvested from three female fish from 18 °C (control) and 24 °C (heat-treated) groups. Tissues were immediately flash frozen in liquid nitrogen and stored at −80 °C until RNA extraction.

### RNA isolation, cDNA synthesis and sequencing

TRIzol reagent (Invitrogen; http://www.invitrogen.com) was used to extract total RNA from liver and head kidney samples. Isolated RNA was checked using a NanoPhotometer spectrophotometer (IMPLEN; http://www.implen.de) and agarose gel electrophoresis. Qubit 2.0 fluorimeter (Life Technologies, ThermoFisher; http://www.thermofisher.com) and a Bioanalyzer 2100 System (Agilent Technologies; http://www.agilent.com) were used to determin the quality and quantity of total RNA. First-strand complementary cDNA was synthesized using a PrimerScript RT Reagent Kit with gDNA Eraser (TaKaRa; http://www.takarabio.com).

Data were collected as previously described in Ma et al. (2020), Specifically after quality control, the rainbow trout reference genome were downloaded from the genome website (https://www.genoscope.cns.fr/trout/) (Berthelot et al., 2014). Index of the reference genome was created using Bowtie v2.2.3, and the sequences were aligned to the reference genome by using TopHat v2.0.1.2 (Trapnell et al., 2012). The FPKM (expected number of fragments per kilobase of transcript sequence per millions base pairs sequence) (He et al., 2015) of each gene was calculated as the unit of expression according to the length of the gene and the result of gene comparison and the fold changes were calculated by using normalized data. Transcripts with absolute fold change value ≥ 1.5, total read number ≥5 and *P* < 0.05 were included in the analyses as significantly differently expressed genes.

16 Hsp70/110 nucleic acid sequences of rainbow trout (*Oncorhynchus mykiss*) were identified as query to compare the high quality data obtained from RNA-Seq. The alignment parameters were set to the maximum of two mismatches.

### Quantitative Real-time PCR

Primer 5 software was used for specific primer design. Our previous experiments have found that *β*-actin (beta-actin) and *EF1*-*α* (elongation factor EF1 alpha) are the most stable reference gene in liver and head kidney of rainbow trout under heat stress, respectively (Ma et al., 2019). Primer sequences of all genes are listed in Table S2. RT-qPCR experiments were performed on a LightCycler 480 Instrument II (Roche; http://www.roche.com). 20 µl reactions contained 2 µl l of diluted cDNA (1:9 dilution), 0.4 µl of each primer, 10 µl of SYBR Premix Ex Taq II (TaKaRa) and 7.2 µl of RNase-free doubledistilled (dd) H_2_O. Cycling sequences were 95 °C for 10 s, followed by 40 cycles at 95 °C for 5 s and 61 °C for 20 s and melting curve analyses at 95 °C for 60 s, 55 °C for 30 s and 95 °C for 30 s. Three biological replicates were employed for each reaction (Ma et al., 2019). Each relative expression of miRNAs was determined using the 2^−ΔΔCt^ method (Livak & Schmittgen, 2001) and subjected to statistical analysis using the SPSS software (SPSS, Chicago, IL, USA).

## Results

### Identification of Hsp70/110 genes in rainbow trout

A total of 16 rainbow trout Hsp70/110 family genes were obtained by homology analysis and named based on the nomenclature of teleost heat shock proteins. They named *hsp70a*, *hsp70b*, *hspa1*, *hspa4* (also known as *HSPH2*), *hspa4L* (also known as *HSPH3*), *hsc70*, *hspa5* (also known as *Grp78*), *hspa5L* (also known as *Grp78L*), *hspa8a* (also known as *hsc71*), *hspa8b*, *hspa9* (also known as *Grp75*), *hspa12a*, *hsp12b*, *hspa13*, *hspa14* (also known as *Hsp70-4*), *hyou1* (also known as *Grp170*) (Table 1). All Hsp70/110 proteins were distributed on chromosomes 12 (*hsp70a, hyou1*), chromosomes 13 (*hsp70b*, *hspa12a*), chromosomes 14 (*hspa4*, *hspa9*), chromosomes 16 (*hspa1*), chromosomes 19 (*hspa4L*), chromosomes 24 (*hsc70*, *hspa13*), chromosomes 29 (*hspa5*,), chromosomes 5 (*hspa5L*), chromosomes 2 (*hspa8b*), chromosomes 9 (*hsp12b*) and chromosomes 10 (*hspa8a*) of rainbow trout, respectively (Table 1). The number of amino acids varies greatly, ranging from 445 to 1,025. Theoretical pI distribution ranged from 4.69 to 7.62, and all of which are acidic (Table 1). The GRAVY of all Hsp70/110 genes identified was negative except for *hspa14* (0.004), that is, they were all hydrophobic proteins (Table 1).

**Table 1:** Physical and chemical property of Hsp70/110 genes in rainbow trout.

Gene	Accession No.	Pr	Chromosomal	Location	Amino acid (aa)	pI	Molecular weight (kDa)	GRAVY	Domain
hspa1	XM_021567143.1	XP_021422818.1	16	67942425–67945641	644	5.46	70.58	−0.422	HSPA1-2_6-8-like_NBD
hsp70a	NM_001124228.1	NP_001117700.1	12	66825718–66829287	644	5.54	71.02	−0.482	HSPA1-2_6-8-like_NBD
hsp70b	NM_001124745.1	NP_001118217.1	13	33931831–33935009	644	5.41	70.99	−0.480	HSPA1-2_6-8-like_NBD
hspa4	XM_021559693.1	XP_021415368.1	14	74147053–74161138	915	4.96	100.2	−0.284	NBD_sugar-kinase_HSP70_actin
hspa4L	XM_021574292.1	XP_021429967.1	19	37480544–37493794	830	5.29	90.07	−0.529	HSPA4_like_NDB
hsc70	XM_021582665.1	XP_021438340.1	24	19720970–19731627	647	5.36	70.85	−0.443	HSPA1-2_6-8-like_NBD
hspa5	XM_021590926.1	XP_021446601.1	29	19957397–19962082	658	4.98	72.55	−0.461	HSPA5-like_NBD
hspa5L	XM_021602455.1	XP_021458130.1	5	27013517–27017496	650	5.19	71.83	−0.452	HSPA5-like_NBD
hspa8a	XM_021617785.1	XP_021473460.1	10	17693103–17713536	647	5.18	70.87	−0.434	HSPA1-2_6-8-like_NBD
hspa8b	XM_021624823.1	XP_021480498.1	2	8546242–8551561	613	5.36	67.80	−0.447	HSPA1-2_6-8-like_NBD
hspa9	XM_021560806.1	XP_021416481.1	14	45830759–45843102	684	5.91	74.11	−0.400	HSPA9-like_NBD
hspa12a	XM_021558936.1	XP_021414611.1	13	62209984–62215546	577	5.58	64.76	−0.394	HSPA12_like_NBD
hspa12b	XM_021614582.1	XP_021470257.1	9	2751625–2764380,	683	7.62	76.40	−0.242	NBD_sugar-kinase_HSP70_actin
hspa13	XM_021582900.1	XP_021438575.1	24	24948380–24953689	445	5.70	48.79.	−0.082	HSPA13-like_NBD
hspa14	XM_021595331.1	XP_021451006.1	unknown	16831–47654	503	6.16	53.72	0.004	HSPA14-like_NBD
hyou1	XM_021623829.1	XP_021479504.1	12	40252422–40280285	1025	5.24	115.0	−0.648	HYOU1-like_NBD

EMBOSS Water software was used to calculate the identity and similarity between protein sequences. The results were found that there were high amino acid identity (>82%) and similarity (>91%) of rainbow trout Hsp70 genes within each group (*hsp70a*, *hsp70b*, *hspa1*, *hsc70*, *hspa9*, *hspa13*, *hspa5*, *hspa14*, *hspa5L*, *hspa8a*, *hspa8b*, *hspa12a* and *hspa12b*), however they share lower amino acid identity (<18%) and similarity (<31%) with Hsp110 genes (*hspa4*, *hspa4L* and *hyou1*) (Tables S3–S4).

### Phylogenetic analysis of rainbow trout Hsp70/110 genes

**Figure 1:**
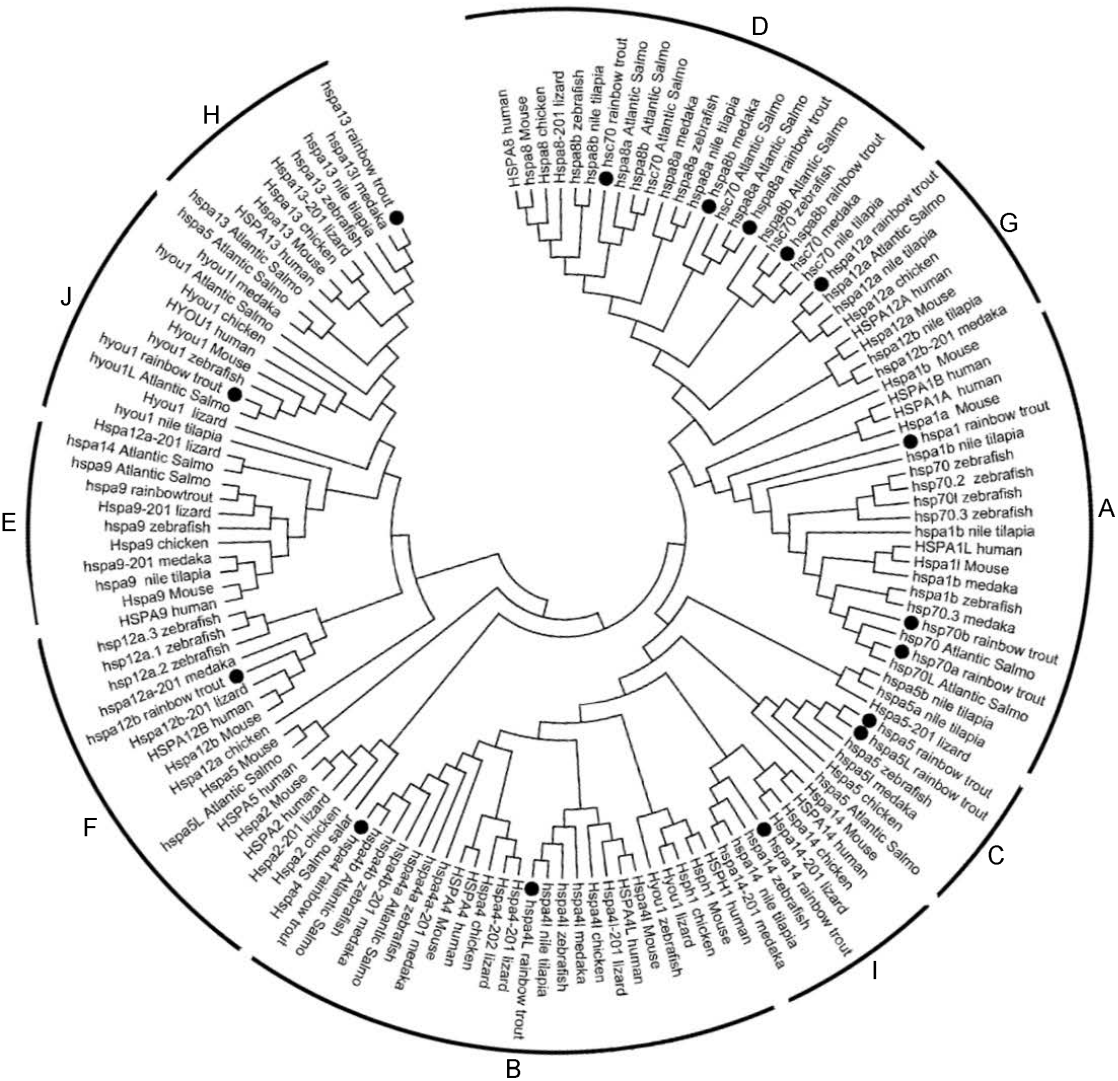
Phylogenetic analysis of the Hsp70/110 proteins form rainbow trout and other species. The phylogenetic tree was constructed by the MEGA 7.0 using the neighbour-joining method. The statistical robustness of the tree was estimated by bootstrapping with 1,000 replicates. Filled circle (∘) represented rainbow trout; The numbers on the nodes indicate the bootstraps value based on 1,000 times replications. The tree was divided into 10 groups (A, C–H, I, J).

Phylogenetic analysis was performed to investigate the phylogenetic relationships of Hsp70/110s with homologs from various species, the phylogenetic tree was constructed using amino acid sequences from rainbow trout, atlantic salmo, zebrafish, medaka, tilapia, fugu, xenopus, turtle, lizard, chicken, turkey, platypus, mouse and human by Neighbor-Joining method. Cluster tree was constructed and divided into 10 groups, among which except for B group, which does not contain Hsp70/110 of rainbow trout, the other 9 groups (A, C–H, I, J) all contain Hsp70/110 of rainbow trout (Fig. 1). In phylogenetic tree, all members of Hsp70/110 of rainbow trout are well separated into distinct systems and they are grouped with genes of other species, with strong bootstrap value.

### Motif and Gene structure analysis of rainbow trout Hsp70/110 genes

**Figure 2:**
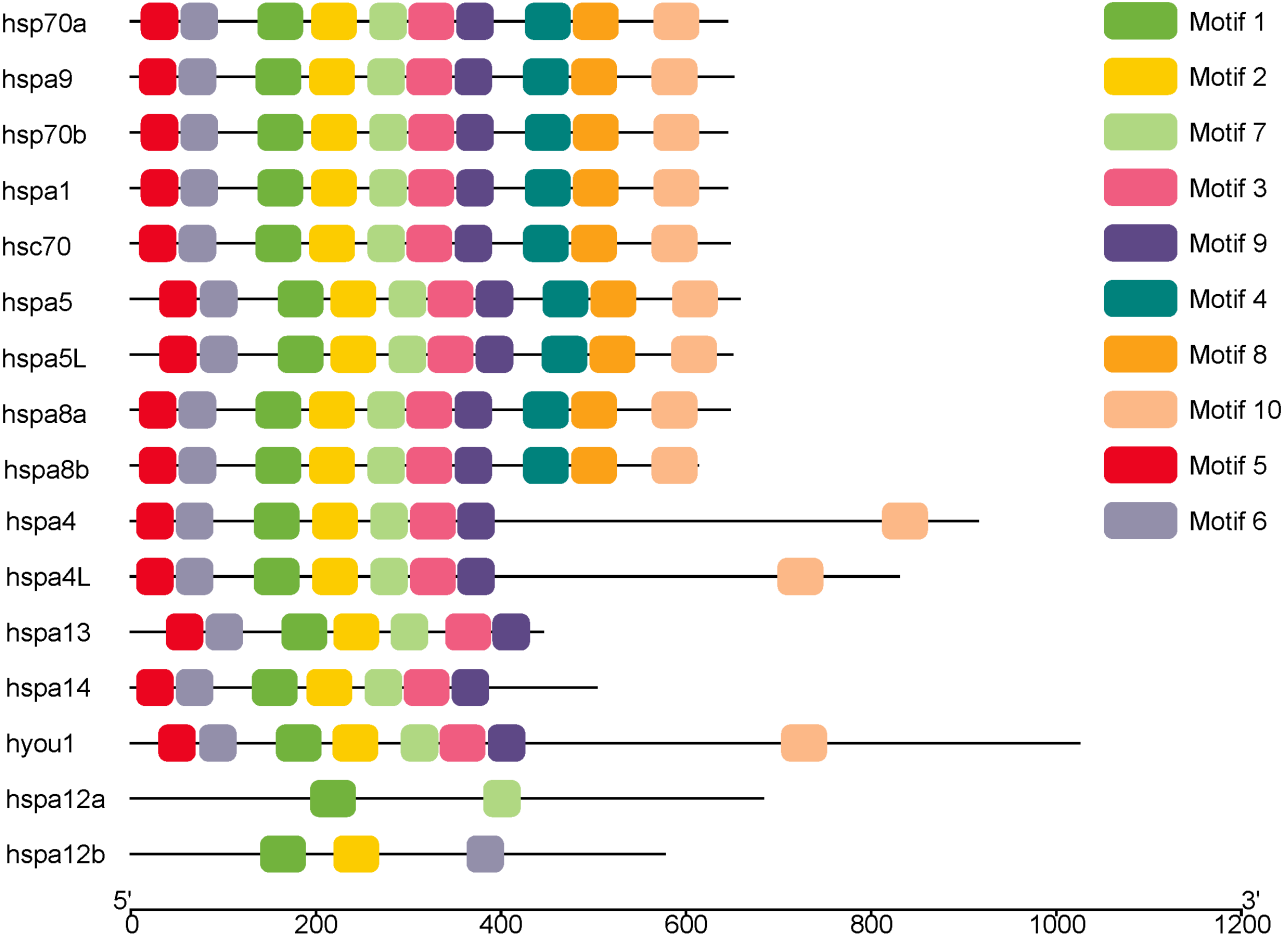
The conserved motifs of the Hsp70/110 proteins according to their phylogenetic relationships. These motifs were identified using Multiple EM for Motif Elicitation (MEME), and the different colors boxes represented different motifs.

MEME was used to analyze the conserved motifs of different groups of protein sequences, and 10 conserved motifs were obtained (Fig. 2), which were named Motif1–Motif10 (Table 2). Although hsp70a, hsp70b, hspa1 hsc70, hspa5, hspa5L, hspa8a, hspa8b and hspa9 belong to different groups, they have the same Motif1–Motif10 of the same kind and distribution. Hspa12a has only two conservative motifs, mitif1 and motif7, and hspa12b has mitif1, motif2 and motif6.

**Figure 3:**
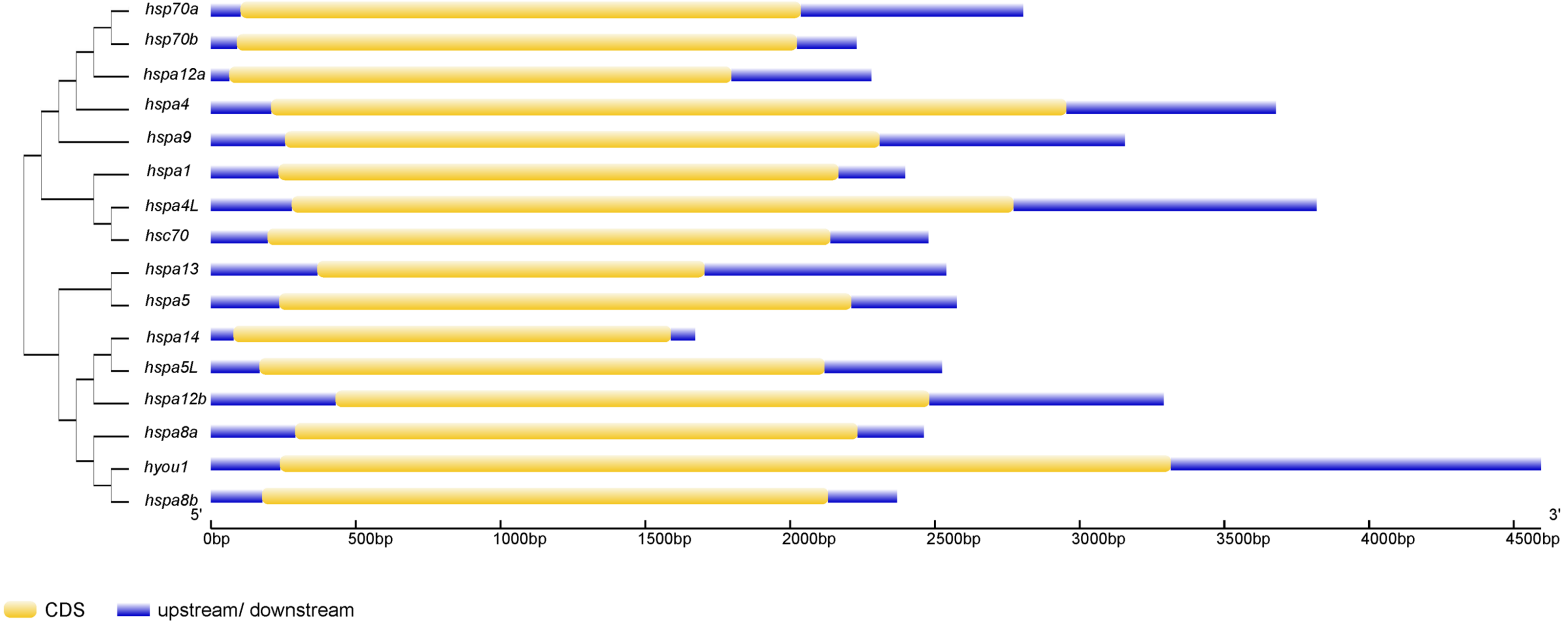
The exon-intron structure of Hsp70/110 genes according to their phylogenetic relationships. The unrooted phylogenetic tree was constructed by the MEGA 7.0 using the neighbour-joining method. The yellow box and violet lines represented CDS and upstream/downstream, respectively. CDS, coding sequences; kp, kilo base pairs.

Phylogenetic trees were constructed using Hsp70/110 protein sequence of rainbow trout and their gene structure was analyzed (Fig. 3). All the 16 Hsp70/110s had only one exon and no intron. The more closely related the genes are, the more similar the genetic structure between them is, for example, hspa4 and hspa4L have smaller differences in length and distribution. The length of Hsp70/110 genes was mostly within 3 KB, and the *hyou1* gene reached 4 KB.

### Subcellular localization and three dimensional structure analysis of Hsp70/110 Proteins

The Hsp70/110 family gene of rainbow trout were expressed in cytoplasm and nucleus, a small amount in extracellular matrix, cytoskeleton and mitochondria, no expression in plasma membrane, and only *hyou1* in lysosomal. The more closely related the genes, the more similar the expression between them, such as *hsp70a* and *hsp70b* are expressed in cytoplasm, nucleus and cytoskeleton (Table 3).

**Figure 4:**
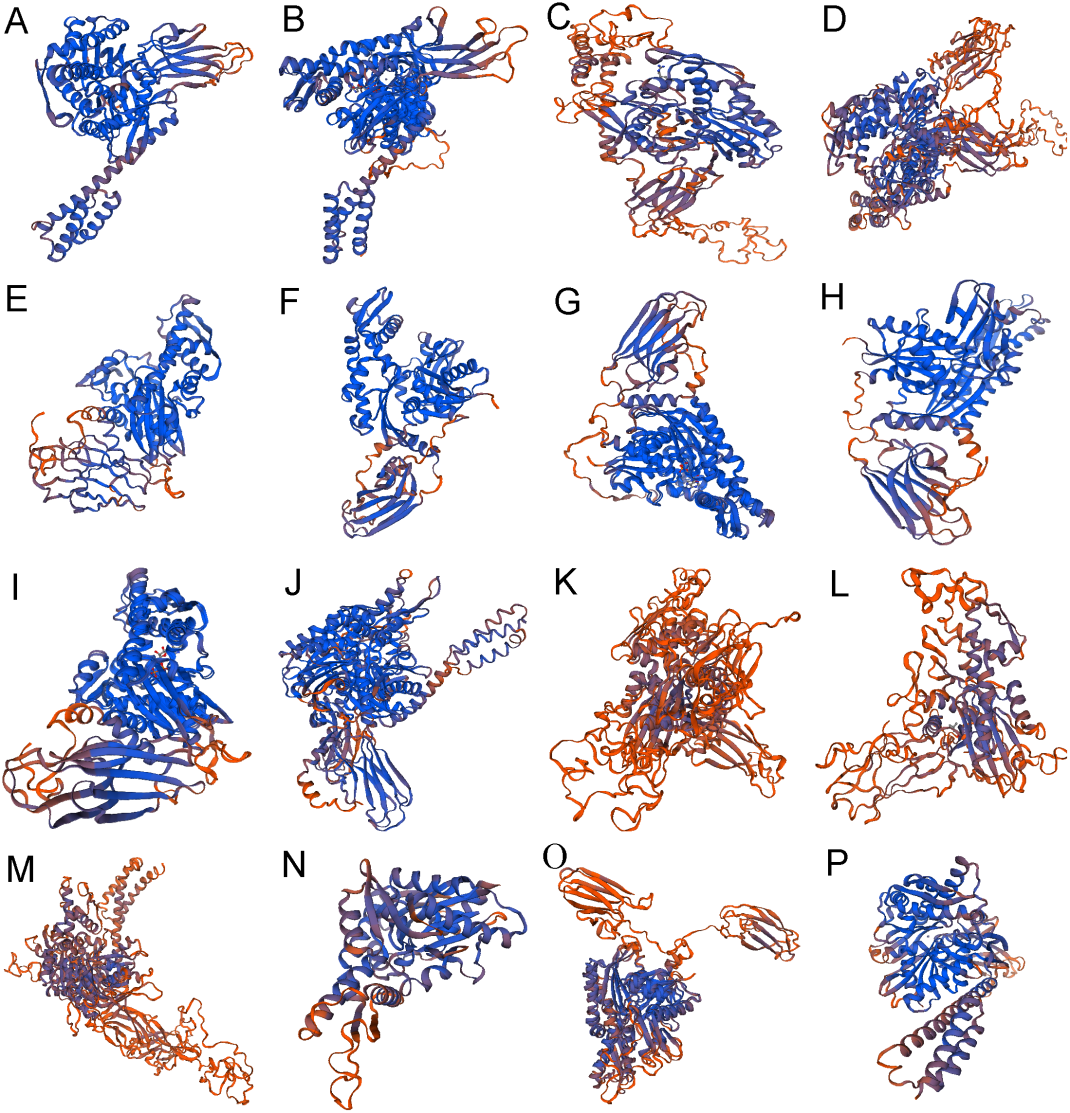
The 3-dimensional structures of Hsp70/110 proteins. The best predicted models were selected with the highest confidence. The secondary structure elements include: alpha helix, beta turn, and random coil. (A) hspa5 (B) hspa5L (C) hspa4 (D) hspa4L (E) hsc70 (F) hspa8a (G) hspa8b (H) hsp70a (I) hsp70b (J) hspa9 (K) hspa12b (L) hspa12a (M) hyou1 (N) hspa13 (O) hspa14 (P) hspa1.

The secondary structure of 16 proteins encoded by the rainbow trout family gene is mainly alpha helix and random coil, among which alpha helix accounts for 28.40% to 46.15%, beta turn accounts for 3.02% to 8.14% and random coil accounts for 29.85% to 40.22% (Table 4) (Fig. 4).

### Analysis of Hsp70/110 genes expression profile in rainbow trout

**Figure 5:**
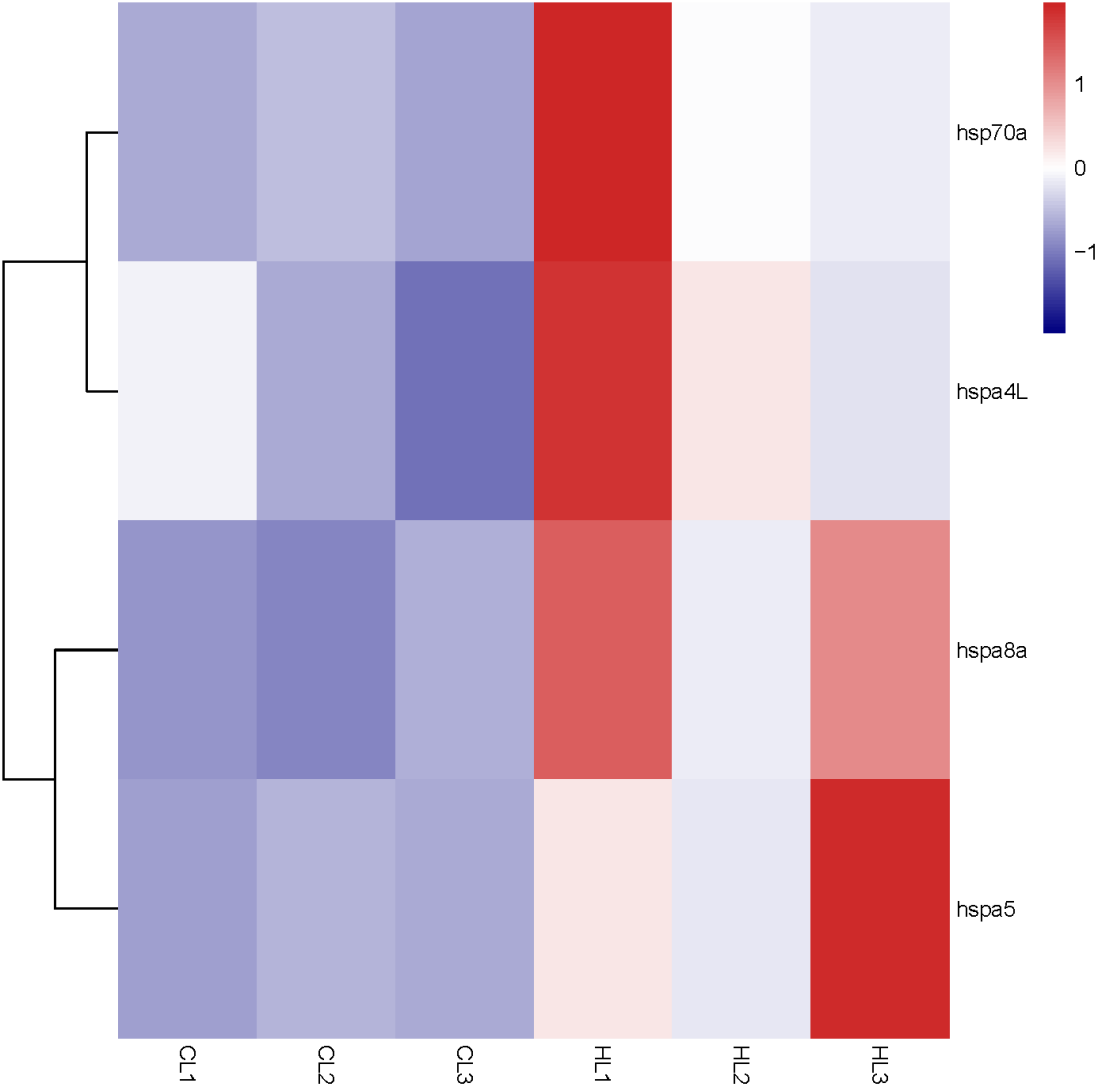
Expression profile of Hsp70/110 genes in liver of rainbow trout in the control group (18 °C) and heat treatment group (24 °C). CL and HL represented control group and heat treatment group, respectively.

**Figure 6:**
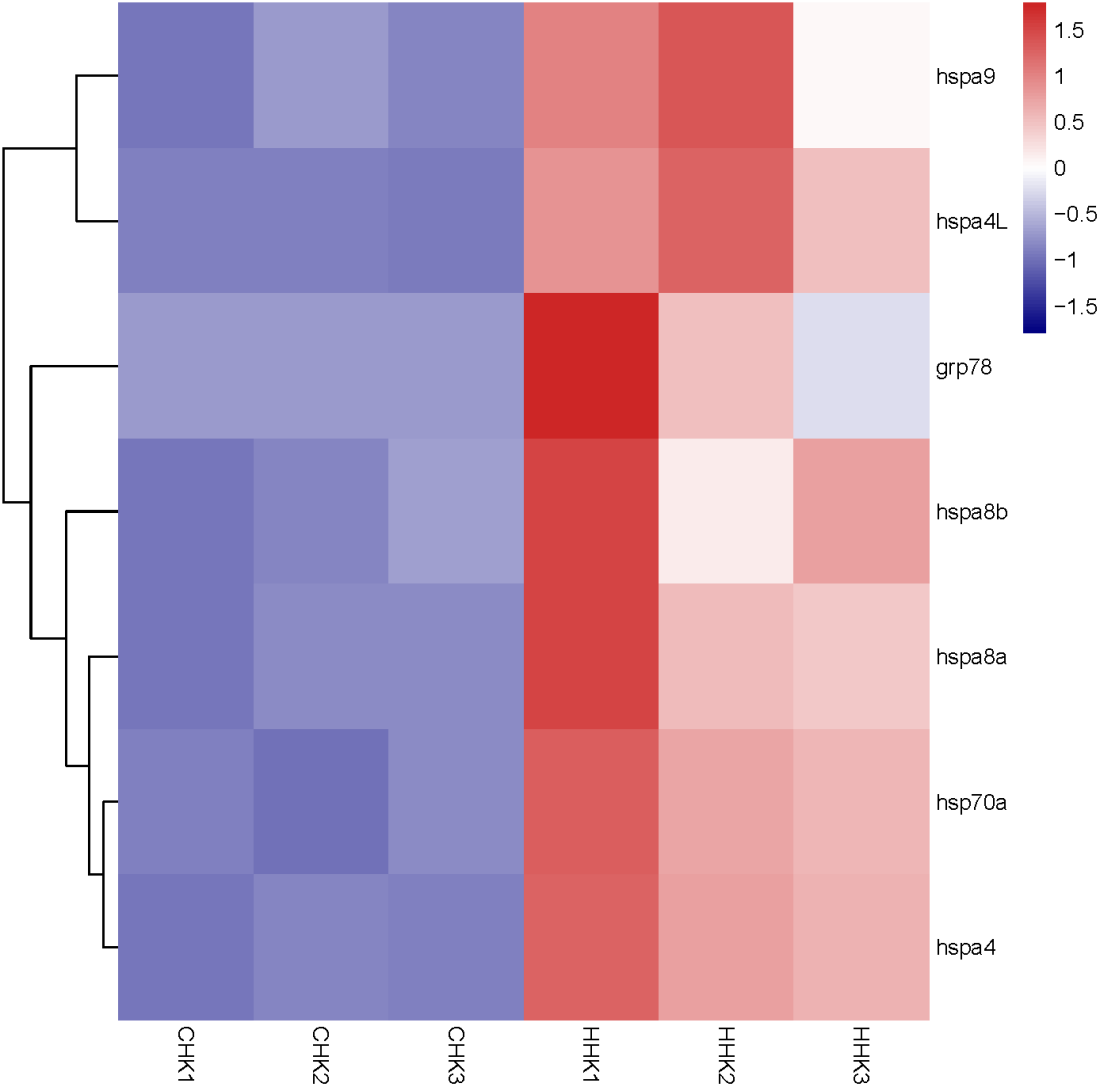
Expression profile of Hsp70/110 genes in head kidney of rainbow trout in the control group (18 °C) and heat treatment group (24 °C). CHK and HHK represented control group and heat treatment group, respectively.

By analyzing the results of our previous RNA-seq data (Table S5), 6 libraries of liver (CL1, CL2, CL3, HL1, HL2 and HL3) and 6 libraries (CK1, CK2, CK3, HK1, HK2 and HK3) of head kidney, the expression levels of 4 genes (*hspa8a*, *hsp70a*, *hspa4L* and *hspa5*) in the liver and 7 genes (*hspa5*, *hspa8b*, *hspa8a*, *hspa4*, *hspa4L*, *hsp70a* and *hspa9*) in the head kidney of the heat treatment group (24 °C) were significantly increased compared with the control group (18 °C) (Figs. 5 and 6). The expression of *hspa8a*, *hspa8b* and *hsp70a* in the liver and head kidney after heat stress was the highest, and they all belonged to the D group. *Hspa5L* is also highly expressed and belongs to the C group. Combined with phylogenetic tree analysis, which can be predicted that C and D groups of Hsp70/110 family mainly play a positive regulatory role in response to heat stress.

**Table 2:** Conserved motifs of Hso70/110 proteins in rainbow trout.

Conserved motif	Length/bp	Amino acid conserved sequence
motif1	50	LGKPVSNAVITVPAYFNDSQRQATKDAGVIAGLNVLRIINEPTAAAIAYG
motif2	50	RNVLIFDLGGGTFDVSILTIEDGIFEVKATAGDTHLGGEDFDNRLVNHFI
motif3	50	TRARFEELNADLFRGTLDPVEKSLRDAKMDKAQIHDIVLVGGSTRIPKIQ
motif4	50	QTQTFTTYSDNQPGVLIQVYEGERAMTKDNNLLGKFELTGIPPAPRGVPQ
motif5	41	IDLGTTYSCVGVFQHGKVEIIANDQGNRTTPSYVAFTDTER
motif6	41	GDAAKNQVAMNPENTVFDAKRLIGRKFDDQVVQSDMKHWPF
motif7	41	NKRAVRRLRTACERAKRTLSSSTQASIEIDSLYEGIDFYTS
motif8	50	VTFDIDANGILNVSAVDKSTGKENKITITNDKGRLSKEDIERMVQEAEKY
motif9	41	LQDFFNGKELNKSINPDEAVAYGAAVQAAILSGDKSENVQD
motif10	50	SDEDKKKILDKCNEVISWLEKNQTAEKEEYEHQQKELEKVCNPIITKLYQ

### Regulated expression of Hsp70/110 genes under heat stress

**Figure 7:**
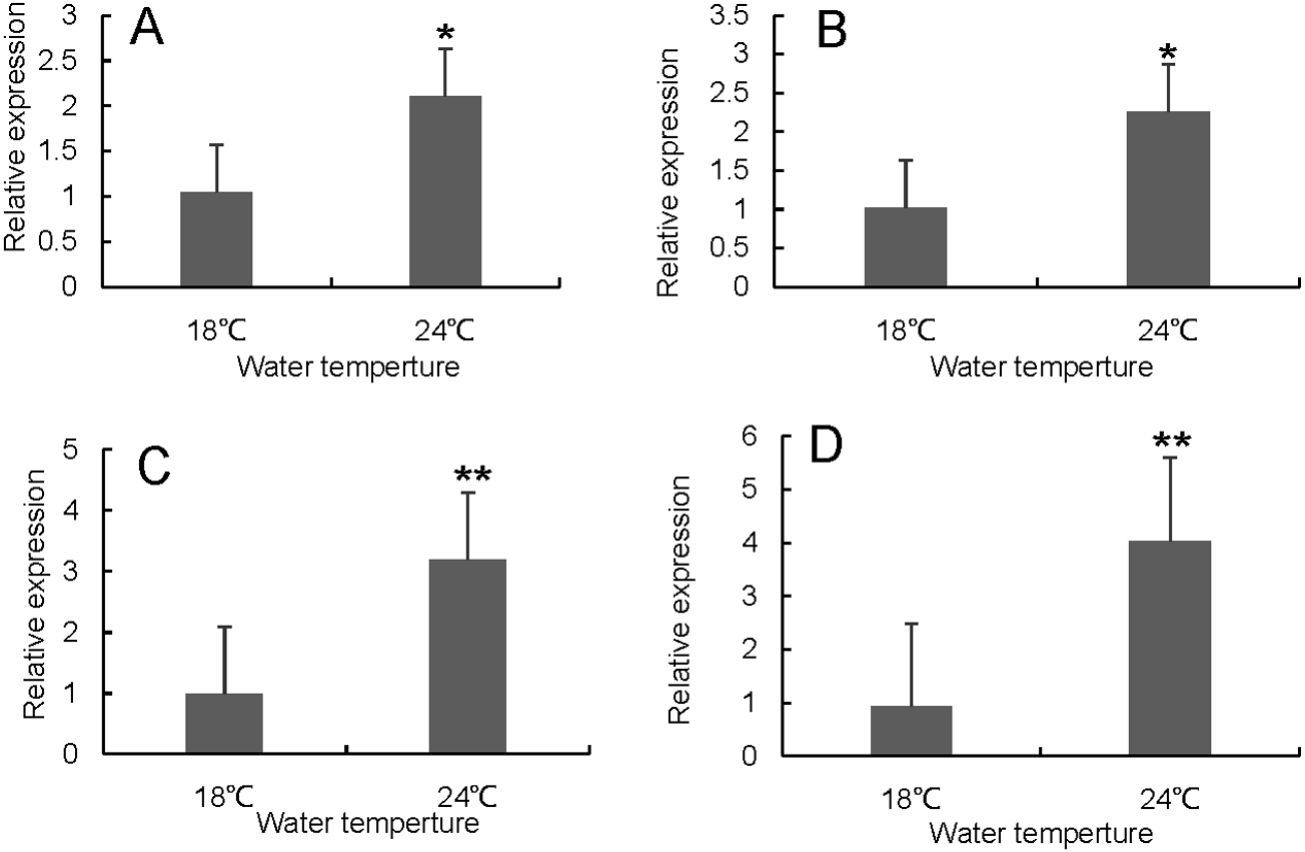
Hsp70/110 gene expression in liver of rainbow trout in the control group (18 °C) and heat treatment group (24 °C). (A) *hsp70a* (B) *hspa5* (C) *hspa4L* (D) *hspa8a*.

**Figure 8:**
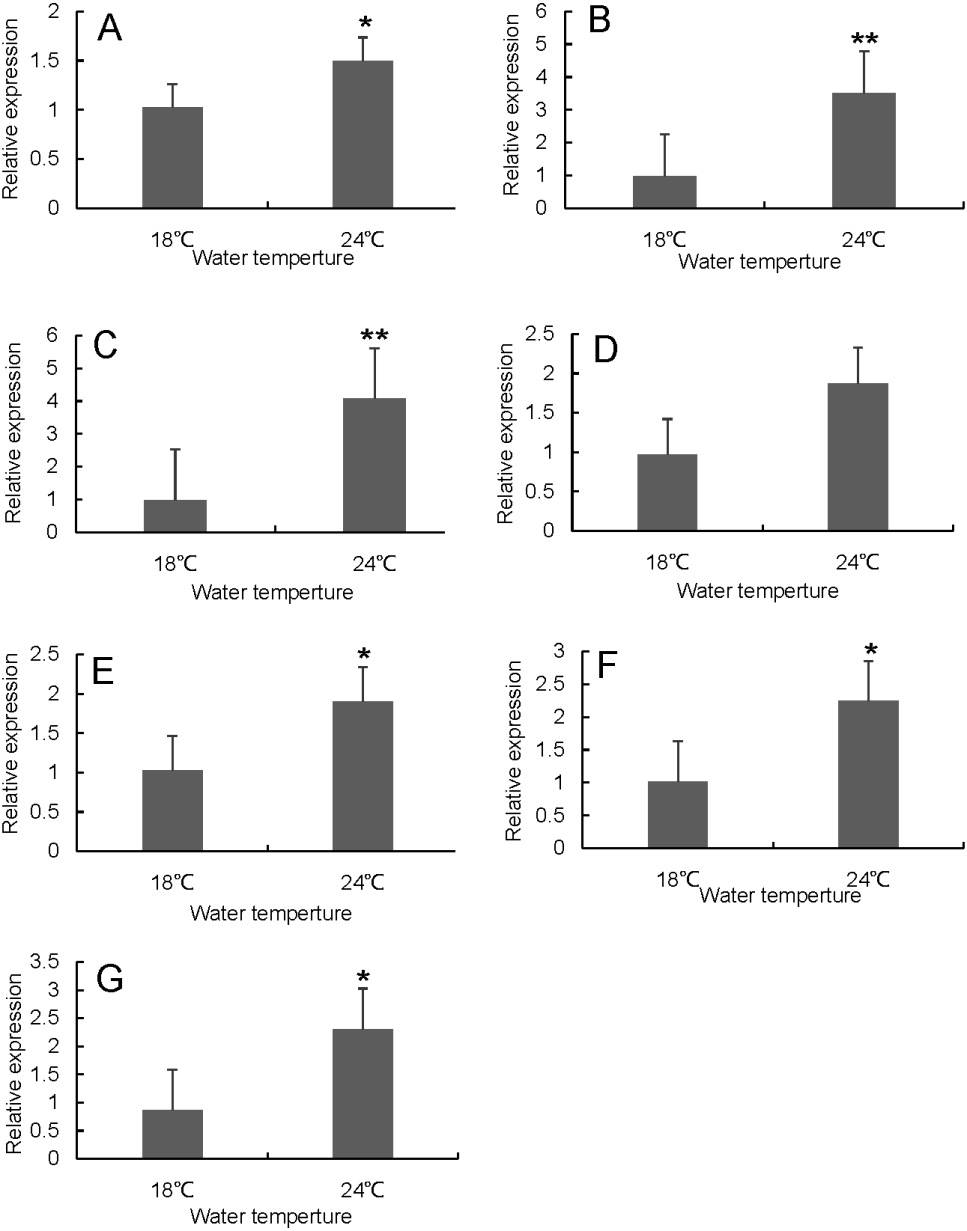
Hsp70/110 gene expression in head kidney of rainbow trout in the control group (18 °C) and heat treatment group (24 °C). (A) *hspa4* (B) *hspa5* (C) *hspa4L* (D) *hspa8b* (E) *hspa8a* (F) *hsp70a* (G) *hspa9*.

RT-qPCR was used to verify the differentially expressed genes in the RNA-seq results of liver and head kidney. The results showed that the RT-qPCR test results were consistent with the RNA-seq results. The expression level of these genes in liver and head kidney increased after heat stress, and the expression level at 24 °C was significantly different from that at 18 °C (Figs. 7 and 8).

**Table 3:** Subcellular location prediction of Hsp70/110 genes in rainbow trout.

Gene	Cytoplasm	Nucleus	Peroxisome	Plasma membrane	Mitochondria
hspa1	19.5	7.5	–	–	–
hsp70a	16	11	–	–	–
hsp70b	17	10	–	–	–
hspa4	5.5	3	–	–	–
hspa4L	20.5	5.5	–	–	5
hsc70	18.5	9.5	–	–	–
hspa5	–	–	4	–	–
hspa5L	–	–	–	–	–
hspa8a	16.5	10.5	–	–	–
hspa8b	18.5	7.5	–	–	–
hspa9	17.25	8.4	–	–	–
hspa12a	15	7	–	–	3
hspa12b	16	10	–	–	–
hspa13	–	–	1.5	–	2.5
hspa14	13	8	–	–	10
hyou1	–	–	–	–	–

Gene	Extracellularpar matrix	Cytoskeleton	Golgi apparatus	Endoplasmic reticulum	Lysosomal
hspa1	–	3	–	–	–
hsp70a	–	3	–	–	–
hsp70b	–	3	–	–	–
hspa4	5	5	–	–	–
hspa4L	–	–	–	–	–
hsc70	–	–	–	–	–
hspa5	14	–	–	12	–
hspa5L	16	–	–	13	–
hspa8a	–	3	–	–	–
hspa8b	–	3	–	–	–
hspa9	–	3	–	–	–
hspa12a	3	–	3	–	–
hspa12b	2	–	–	–	–
hspa13	6	–	–	17	–
hspa14	–	–	–	–	–
hyou1	12	–	5.5	–	5

**Notes.**

The value is classifying sorting signals amino acid composition and functional motifs. “–” is used to signify the absence of a value.

**Table 4:** The secondary structure of Hsp70/110 proteins sequence in rainbow trout.

Protein	Alpha helix (%)	Beta turn (%)	Random coil (%)
hspa1	41.61	7.30	33.23
hsp70a	41.93	7.14	32.30
hsp70b	41.46	7.30	33.07
hspa4	40.66	4.15	40.22
hspa4L	43.01	3.37	39.52
hsc70	42.04	7.11	32.77
hspa5	43.47	7.60	30.40
hspa5L	43.38	7.38	30.46
hspa8a	42.66	7.11	31.99
hspa8b	44.37	6.85	29.85
hspa9	41.52	5.89	33.45
hspa12a	34.14	6.07	37.61
hspa12b	28.40	4.83	45.83
hspa13	40.00	6.25	32.13
hspa14	36.98	5.57	34.79
hyou1	46.15	3.02	39.71

## Discussion

Heat shock protein (Hsps) are highly conserved protein widely found in prokaryotes and eukaryotes. These proteins play an important role in providing heat resistance by maintaining protein homeostasis and have also been found to be induced in other abiotic stresses in animals. To survive, an organism must be able to maintain intracellular homeostasis in a constantly changing environment. Molecular chaperones are crucial to this effort because they provide a “buffer” that helps protect cellular proteins from extreme conditions such as sudden temperature rises, oxidative stress, heavy metal exposure, oxygen deprivation and metabolic disorders (Craig, 2018; Fernandez-Fernandez et al., 2017; Nillegoda, Wentink & Bukau, 2018; Mogk et al., 1999). Hsp70/110s is a molecular chaperone that enhances the response of cells to various stress stimuli such as heat shock (Gao et al., 2018). Recent research has made significant progress in better understanding the mechanisms of action of Hsp70 and its co-partners, providing insights into the structural and functional characteristics of different members of the network. Genome-wide identification and annotation of Hsp70/110 gene family has been performed in some teleost, including *Boleophthalmus pectinirostris* (Deng et al., 2019)*, Paralichthys olivaceus* (Liu et al., 2019), *Larimichthys crocea* (Xu, Xu & Han, 2018), and channel catfish (Song et al., 2016). At the same time identification of the Hsp70/110 gene family has been performed in invertebrates, such as sea cucumber (Gao et al., 2018). However, there is still a very limited genome-wide identification and annotation of Hsp70/110 genes in rainbow trout. Therefore, we performed an overall analysis of the hsp70/110s gene family in the rainbow trout, including an analysis of the phylogeny, gene structure, conserved motifs, and expression patterns under heat stress. These results will provide further insights into the evolutionary profile of Hsp70/110 in fish species and provide insights into their role in the study of fish stress resistance.

Members of this Hsp70/110 family play key roles in the folding of newly synthesized proteins, stabilization and refolding of misfolded proteins, dissolution of protein aggregates, transport of proteins, and proteolytic degradation of unstable proteins. The involvement of Hsp70 in many protein-folding activities related to housekeeping and stress not only highlights the critical importance of these chaperones in maintaining protein homeostasis, but also links them to many pathophysiological conditions in humans (Qu et al., 2015). Hsp70 is one of the most abundant chaperones in cells, accounting for 0.5–2% of the total protein mass of cells (Finka et al., 2015). In the present study, a total of 16 Hsp70/110 gene family were identified. In terms of the number and members of Hsp70/110 gene family, there are some differences between the teleost analyzed. Compared with humans and other fish species, most of Hsp70/110 were found in rainbow trout, but as in the study of channel catfish (Song et al., 2016), *hsph1* was not found in the genome of rainbow trout, except for zebrafish genome, no *hsph1* was found in all other fish genome. Sequence analysis showed that there were two copies of *hspa8* (*hspa8a* and *hspa8b*), *hspa4* (*hspa4* and *hspa4L*), *hspa5* (*hspa5* and *hspa5L*) and *hspa12* (*hspa12a* and *hspa12b*), one copy of *hspa13* and *hspa14* in the rainbow trout, which may be because the genome of rainbow trout has also experienced whole genome replication (WGD) (Berthelot et al., 2014). Salmon have experienced four whole-genome duplication events in their evolutionary history, namely 1R and 2R, sequentially occurring from chordate ancestors to jawed and possibly jawed/cyclostome vertebrates (Macqueen, Garcia de la Serrana & Johnston, 2013). 3R, which was later experienced by a common bony fish ancestor (Macqueen, Garcia de la Serrana & Johnston, 2013), and 4R, all existing salmon are derived from 4R (Macqueen, Garcia de la Serrana & Johnston, 2013). The 4R of salmonid fish results in expansions to existing vertebrate gene families and many types of functional divergence (Macqueen, Garcia de la Serrana & Johnston, 2013). Gene duplication not only expands the genome content, but also diversifies the functions of genes to ensure the best adaptability and evolution. After the teleost underwent TSGD event, lineage-specific gene duplication and loss are frequently observed during evolution, which provides useful information for the study of gene evolutions (Niimura, Matsui & Touhara, 2014; Jiang et al., 2016). Duplication events occur between different chromosomes, suggesting that repetitive events may play an important role in gene amplification in the Hsp70/110 family gene expansion.

Hsp70s are made up of ATP-dependent N-terminal ATPase domain (NBD), substrate binding domain and C-terminal polytropic domain (SBD) (Mayer & Bukau, 2005). ATPase domain interacts with ATP and regulates the functional domain of substrate interaction, namely, the expansion of variable domain, exposure of substrate binding domain, binding with substrate, and covering the variable domain, as a molecular chaperone involved in protein folding or targeted degradation (Marcinowski et al., 2011).Through motif analysis, which was found that the structure of hspa12 gene was very different from that of other members of the Hsp70/110 family. Only motif 1 and motif7 were found in hspa12a, and only motif 1, motif 2 and motif 7 were found in hspa12b, because hspa12 had atypical ATPase domain (Han et al., 2003). The difference of gene structure may be related to the different functions of biological process (Zhang et al., 2013). Intron deletion is considered to be a typical feature of most Hsp genes. So far, there is no conclusive experimental evidence to prove the role of gene structure and Hsp gene expression. On the one hand, before translation, Hsps mRNA was excluded from the stress particles and given priority to translation when pre-mRNA splicing and global hat-dependent translation were inhibited due to heat stress. After translation, the Hsps protein is thermodynamically stable at elevated temperatures. To reduce the heat shock response and prevent subsequent damage, intronless Hsps mRNA avoids various decay pathways and allows partner activity to start early after synthesis (Evgen’ev, Garbuz & Zatsepina, 2014). On the other hand, some studies have shown that genes with introns allow more mRNA accumulation than genes without introns (Le Hir, Nott & Moore, 2003). In this study, there is no intron in the Hsp70/110 genes of all rainbow trout, which may indicate that the functional conservation of this gene family.

Hsp70 levels are tightly regulated according to cell needs such as growth or tissue-specific function, and Hsp70 family members are present in most cell compartments (cytoplasm, nucleus, endoplasmic reticulum, mitochondria, chloroplasts). In this study, The Hsp70 family genes of rainbow trout were mainly expressed in Cytoplasm, Nucleus and Extracellular matrix (Table 3).

The change of Hsp70 expression under various stress conditions has been a hot spot in the study of fish heat shock protein. The expression patterns of *Brachydanio Rerio* were different under heat stress and cold stress. Hsp70 expression was up-regulated under heat stress and stable under cold stress (Airaksinen et al., 2003). The expression of Hsp70 in *Cyprinus Carpio* under heat stress is tissue specific, and the expression level in liver is significantly higher than that in gills and heart, and increases steadily with the increase of temperature (Das, Gupta & Manna, 2005). The two Hsp70 subtypes, Hsp70-1 and Hsp70-2, were significantly different in expression changes during heat stress. Hsp70-2 was induced earlier and its expression level was higher than that of Hsp70-1 (Metzger, Healy & Schulte, 2016). Although some studies have reported the expression of one Hsp70 gene in rainbow trout under heat stress (Xia et al., 2018), the participation of Hsp70 in the response to heat stress in this species has not been systematically analyzed. Hsp70/110 has been shown to have general cellular protective properties by promoting increased anti-apoptotic activity through overexpression, thereby reducing the effects of stress and injurious stimuli (Tranter et al., 2011; Gabai et al., 1997). In this study, we analyzed the RNA-seq data of our team in the early stage and found that 4 Hsp70/110 family genes in the liver (*hsp70a*, *hspa8a*, *hspa5* and *hspa4L*) and 8 Hsp70/110 family genes *hsp70a*, *hspa4*, *hspa8a*, *hspa8b*, *hspa4L*, *hspa5* and *hsp70b*) in the head kidney were significantly up-regulated after thermal stimulation, and verified by RT-qPCR, and the results of RNA-seq and RT-qPCR were consistent. The expression of hspa5 gene under heat stress was induced by temperature. Previous studies have shown this to be true. The mRNA expression of hspa5 in rainbow trout at 25 °C and 26 °C was significantly higher than that at 18 °C (*P* < 0.05) (Xia et al., 2018).

Cellular stress caused by the accumulation of misfolded proteins in the endoplasmic reticulum (ER) activates a complex signaling network known as the unfolded protein response (UPR).

The metabolism of hypothermic fish is highly dependent on the ambient temperature under stress, and different tissues adopt different metabolic strategies. *Hyou1* is a hypoxic up-regulated protein, and its up-regulation in the head kidney confirmed a positive correlation between hypoxia and high temperature (Pörtner & Peck, 2010), indicating that rainbow trout subjected to heat stress suffered damaging aerobic metabolism (Xu et al., 2016). The relationship between temperature and oxygen saturation of *Carassius carassius* (Rissanen et al., 2006), *Atlantic salmon* (Olsvik et al., 2013) and *Cynoglossus semilaevis* (Guo et al., 2016) was also be found. Besides being used as biomarkers of physiological stress response (Beg et al., 2010; Wang et al., 2007), Hsp70/110 is also involved in the immune response (Vabulas et al., 2002). Cellular immune response is the main overexpressed physiological function of the head kidney under environmental stress (Verleih et al., 2015). As one of the major lymphoid tissues in fish, the head kidney can carry out humoral and cellular immune responses. In this study, we identified 7 Hsp70/110 genes in the head kidney that were significantly up-regulated under heat stress. The main molecular functions of Major Histocompatibility Complex (MHC) include the processing and presentation of protein antigens, the extensive involvement in the induction and regulation of immune responses, which plays a key role in vertebrate adaptation and innate immunity (Horton et al., 2004). Hsp70, encoded by the HSPA1B gene, is located in MHC class III (Pistono et al., 2020). MHC related gene expression was up-regulated in rainbow trout head kidney at high temperature, indicating that Hsp70/110 genes was involved in immune response. Four Hsp70/110 genes (*hsp70a*, *hspa5*, *hspa4L* and *hspa8a*) were significantly up-regulated in both the liver and the head kidney, and these co-expressed genes confirmed the important regulatory role of heat stress in different tissues. Tissue-specific expression profiles were observed among the two tissues analyzed in this study. Three Hsp70/110 genes (*hsp70b*, *hspa8b* and *hspa9*) that were significantly up-regulated only in the head kidney, but showed no significant differences in the liver, which may indicate that the gene had some tissue specificity.

## Conclusion

A total of 16 Hsp70/110s have been identified and characterized from the genome an RNA-seq data of rainbow trout. The gene structure, conserved protein domain and phylogenetic relationship of these Hsp70/110 genes were systematically analyzed, which provided a strong support for the classification of Hsp70/110s genes. The expression levels of rainbow trout Hsp70/110 genes in liver and head kidney was investigated. The results indicated that seven out of 16 Hsp70/110 genes were up-regulated in head kidney and four out of 16 Hsp70/110 genes were up-regulated in liver under heat stress, which indicated that Hsp70/110 gene family was involved in the heat stress process of rainbow trout. Our study comprehensively reviewed the Hsp70/110 gene family of rainbow trout, provided a new insight into the evolution of the gene family of teleost.

##  Supplemental Information

10.7717/peerj.10022/supp-1 Supplemental Information 1 All Species accession numbers of hsp70/110

10.7717/peerj.10022/supp-2 Supplemental Information 2 Primers used for the quantitative real-time PCR (RT-qPCR)

10.7717/peerj.10022/supp-3 Supplemental Information 3 Protein sequence identity of Hsp70/110 genes

10.7717/peerj.10022/supp-4 Supplemental Information 4 Protein sequence similarity of Hsp70/110 genes

10.7717/peerj.10022/supp-5 Supplemental Information 5 RNA-Seq Expression Data

10.7717/peerj.10022/supp-6 Supplemental Information 6 Raw data

## Additional Information and Declarations

### Data Availability

The following information was supplied regarding data availability: The raw measurements are available as a Supplemental File.
