# Supplementary material for: Genome-wide identification of Hsp70/110 genes in rainbow trout and their regulated expression in response to heat stress
Source: PeerJ. 2020 Oct 23;8:e10022. doi: 10.7717/peerj.10022 (PMC7587057; doi:10.7717/peerj.10022)
Supplement: Supplemental Information 1 [file peerj-08-10022-s001.docx]

**Table S1 All Species accession numbers of hsp70/110**

| No. | Species name | **Associated Gene Name** | Protein ID |
| --- | --- | --- | --- |
| 1 | *Homo sapiens* | HSPA1A | NP_005336.3 |
|  |  | HSPA1B | NP_005337.2 |
|  |  | HSPA1L | NP_005518.3 |
|  |  | HSPA2 | NP_068814.2 |
|  |  | HSPA4 | NP_002145.3 |
|  |  | HSPA4L | NP_055093.2 |
|  |  | HSPA5 | NP_005338.1 |
|  |  | HSPA6 | NP_002146.2 |
|  |  | HSPA7 | UniProtKB/Swiss-Prot: P48741.2 |
|  |  | HSPA8 | NP_006588.1 |
|  |  | HSPA9 | NP_004125.3 |
|  |  | HSPA12A | NP_079291.2 |
|  |  | HSPA12B | NP_443202.3 |
|  |  | HSPA13 | NP_008879.3 |
|  |  | HSPA14 | NP_057383.2 |
|  |  | HSPH1 | NP_006635.2 |
|  |  | HYOU1 | NP_001124463.1 |
| 2 | *Mus musculus* | Hspa1a | NP_034609.2 |
|  |  | Hspa1b | NP_034608.2 |
|  |  | Hspa1l | NP_038586.2 |
|  |  | Hspa2 | NP_001002012.1 |
|  |  | Hspa4 | NP_032326.3 |
|  |  | Hspa4l | NP_035150.3 |
|  |  | Hspa5 | NP_001156906.1 |
|  |  | hspa8 | NP_112442.2 |
|  |  | Hspa9 | NP_034611.2 |
|  |  | Hspa12a | NP_780408.1 |
|  |  | Hspa12b | NP_082582.1 |
|  |  | Hspa13 | NP_084477.1 |
|  |  | Hspa14 | NP_056580.2 |
|  |  | Hsph1 | NP_038587.2 |
|  |  | Hyou1 | NP_067370.3 |
| 3 | *Gallus gallus* | Hspa2 | NP_001006686.1 |
|  |  | Hspa4 | XP_003642142.1 |
|  |  | Hspa4l | NP_001012594.1 |
|  |  | Hspa5 | NP_990822.1 |
|  |  | Hspa8 | NP_990334.1 |
|  |  | Hspa9 | NP_001006147.1 |
|  |  | Hspa12a | XP_421779.3 |
|  |  | Loc770082 | XP_001233402.2 |
|  |  | Hspa13 | NP_001025964.2 |
|  |  | Hspa14 | XP_416996.3 |
|  |  | Hsph1 | NP_001153170.1 |
|  |  | Hyou1 | NP_001006588.1 |
| 4 | *Anolis carolinensis* | Hspa2-201 | ENSACAP00000015494 |
|  |  | Hspa4-201 | ENSACAP00000013089 |
|  |  | Hspa4-202 | ENSACAP00000023153 |
|  |  | Hspa4l-201 | ENSACAP00000011642 |
|  |  | Hspa5-201 | ENSACAP00000004078 |
|  |  | Hspa8-201 | ENSACAP00000004798 |
|  |  | Hspa9-201 | ENSACAP00000015698 |
|  |  | Hspa12a-201 | ENSACAP00000009931 |
|  |  | Hspa12b-201 | ENSACAP00000004138 |
|  |  | Hspa13-201 | ENSACAP00000000965 |
|  |  | Hspa14-201 | ENSACAP00000001088 |
|  |  | Hyou1 | ENSACAP00000013983 |
|  |  | Hsph1 | ENSACAP00000004913 |
| 5 | *Danio rerio* | hsp70.3 | NP_571472.1 |
|  |  | hsp70.2 | XP_003198158.1 |
|  |  | mcm5(hsp70) | ENSDARP00000109199 |
|  |  | LOC798846(hspa1b) | NP_001093532.1 |
|  |  | hsp70l | NP_001107061.1 |
|  |  | hspa4a | NP_999881.1 |
|  |  | hspa4b | NP_956151.1 |
|  |  | wu:fc07b10(hspa4l) | XP_690505.2 |
|  |  | hspa5 | NP_998223.1 |
|  |  | hspa8a | NP_001103873.1 |
|  |  | hspa8b[hsc70.2(LOC562935)] | NP_001186941.1 |
|  |  | hsc70 | NP_956908.1 |
|  |  | hspa9 | NP_958483.2 |
|  |  | si:dkey-61p9.8 (hsp12a.1) | NP_001038342.1 |
|  |  | si:dkey-61p9.4(hsp12a.2) | XP_003198604.1 |
|  |  | si:dkey-61p9.6(hsp12a.3) | NP_001038346.2 |
|  |  | hspa13 | NP_001082948.1 |
|  |  | hspa14 | NP_001038541.1 |
|  |  | LOC557824 | XP_001919957.1 |
|  |  | hyou1 | NP_997868.1 |
| 6 | *Oryzias latipes* | hsp70.3(hspa) | XP_004071143.1 |
|  |  | hsp70-5(hspa1b) | NP_001098384.1 |
|  |  | hsc70 | NP_001098385.1 |
|  |  | hsc70.2(hspa8b) | XP_004075396.1 |
|  |  | hspa8a | UniProtKB/Swiss-Prot: Q9W6Y1.1 |
|  |  | hspa4a-201 | ENSORLP00000001795 |
|  |  | hspa4b-201 | ENSORLP00000007499 |
|  |  | hspa4l | XP_004082341.1 |
|  |  | hspa5l | XP_004074796.1 |
|  |  | hspa9-201 | ENSORLP00000013340 |
|  |  | hspa12a-201 | ENSORLP00000001447 |
|  |  | hspa12b-201 | ENSORLP00000007349 |
|  |  | hspa13l | XP_004075919.1 |
|  |  | hspa14-201 | ENSORLP00000015785 |
|  |  | hyou1l | XP_004084567.1 |
| 7 | *oreochromis niloticus* | hspa1lpartial(hsp70.3) | xp_003442504.1 |
|  |  | loc100704606 (hspa1b) | xp_003444871.1 |
|  |  | hspa8a | xp_003448938.1 |
|  |  | hsc70 | xp_003454400.1 |
|  |  | hspa8b | xp_003455104.1 |
|  |  | hspa4l | xp_003453147.1 |
|  |  | hspa5a | xp_005470418.1 |
|  |  | hspa5b | xp_003459659.1 |
|  |  | hspa9 | xp_003459471.1 |
|  |  | loc100699432 | xp_003457416.1 |
|  |  | hspa12b | xp_003452414.1 |
|  |  | loc100708509 | xp_003441638.1 |
|  |  | loc100697637 | xp_003455685.1 |
|  |  | loc100691644 | xp_003448981.1 |
| 8 | Salmo salar | Hsp70 | APP91800.1 |
|  |  | Hsc70 | XP_013992647.1 |
|  |  | Hspa1 | XP_014046746.1 |
|  |  | Hspa4 | ACN58679.1 |
|  |  | Hspa4L | XP_013992269.1 |
|  |  | Hspa5 | XP_013991602.1 |
|  |  | Hspa5L | XP_014053074.1 |
|  |  | Hspa8a | XP_014018258.1 |
|  |  | Hspa8b | XP_014053073.1 |
|  |  | Hspa9 | ACN60239.1 |
|  |  | Hsp12a | XP_014058216.1 |
|  |  | Hsp12b | XP_014012126.1 |
|  |  | Hspa13 | XP_014070433.1 |
|  |  | Hspa14 | ACN58674.1 |
|  |  | Hyou1 | XP_013992641.1 |
