# Supplementary material for: Genome-wide identification of Hsp70/110 genes in rainbow trout and their regulated expression in response to heat stress
Source: PeerJ. 2020 Oct 23;8:e10022. doi: 10.7717/peerj.10022 (PMC7587057; doi:10.7717/peerj.10022)
Supplement: Supplemental Information 2 [file peerj-08-10022-s002.docx]

**Table S2.** Primers used for the quantitative real-time PCR (RT-qPCR)

| Gene Name | Accession No. | Primer Sequence (5'to3') |
| --- | --- | --- |
| *hsp70a* | NM_001124228.1 | F: 5'-CTGCCTGTGAGCGTGCCAAG-3' |
|  |  | R: 5'-CAAAGCGAGCCCTGGTGATGG-3' |
| *hspa4* | XM_021559693.1 | F: 5'- AGAAGGACATCAGCGGCAACAAG-3' |
|  |  | R: 5'- TGTAGAAGTCGGCACCCTCGTAG-3' |
| *hspa4L* | XM_021574292.1 | F: 5'-AGGACCTGCTGCTGCTGGAC-3' |
|  |  | R: 5'- GGCTGGTTGTCTGAGTATGTGGTG-3' |
| *hspa5* | XM_021590926.1 | F: 5'- AGGCTCATTGGTGATGCTGCTAAG-3' |
|  |  | R: 5'-CCACGGTATCCTCAAACCTTCTGC-3' |
| *hspa8a* | XM_021617785.1 | F: 5'- CCTCCATCACCAGGGCTCTCC-3'  R: 5'- GCTCTTGTCCACCGCTGCTAC-3' |
| *hspa8b* | XM_021624823.1 | F: 5'- TGGTGGGAATTGATGTGGGCTTTC-3' |
|  |  | R: 5'-GGTGTACTTCGGTCGCTGTATTCG-3' |
| *hspa9* | XM_021560806.1 | F: 5'-GCCTGAGGACATTGAGCGGATG-3' |
|  |  | R: 5'-GATTCTTGAGCGAGTAGGCGTAGC-3' |
| *β-actin* | AJ438158.1 | F: 5'- TGGACTTTGAGCAGGAGATGG-3' |
|  |  | R: 5'- AGGAAGGAGGGCTGGAAGAG-3' |
| *EF1-α* | AF498320.1 | F: 5'- GCTGGTTCAAGGGATGGAAG-3' |
|  |  | R: 5'- ACGAAGGGGCTTGTCTGTG-3' |

**F,** forward; **R**, reverse
