# Supplementary material for: Genome-wide identification of Hsp70/110 genes in rainbow trout and their regulated expression in response to heat stress
Source: PeerJ. 2020 Oct 23;8:e10022. doi: 10.7717/peerj.10022 (PMC7587057; doi:10.7717/peerj.10022)
Supplement: Supplemental Information 3 [file peerj-08-10022-s003.docx]

**Table S3 Protein sequence identity of Hsp70/110 genes**

| aa% Identity | hsp70a | hsp70b | hspa4 | hspa1 | hspa4L | hsc70 | hspa13 | hspa5 | hspa14 | hspa5L | hspa8a | hspa8b | hspa9 | hspa12a | hspa12b | hyou1 |
| --- | --- | --- | --- | --- | --- | --- | --- | --- | --- | --- | --- | --- | --- | --- | --- | --- |
| hsp70a | 100.0 | 98.1 | 32.4 | 87.6 | 29.5 | 83.3 | 41.2 | 61.6 | 36.1 | 61.9 | 83.6 | 85.1 | 49.1 | 20.6 | 19.2 | 25.6 |
| hsp70b |  | 100.0 | 32.7 | 87.5 | 29.7 | 83.1 | 41.2 | 61.6 | 35.9 | 61.9 | 83.4 | 84.9 | 49.6 | 20.3 | 20.1 | 26.0 |
| hspa4 |  |  | 100.0 | 33.7 | 52.0 | 31.3 | 27.3 | 31.6 | 31.3 | 31.4 | 31.8 | 32.5 | 48.6 | 20.7 | 18.7 | 24.5 |
| hspa1 |  |  |  | 100.0 | 26.5 | 84.2 | 42.4 | 62.8 | 36.1 | 62.6 | 84.2 | 84.8 | 31.6 | 19.8 | 20.6 | 25.3 |
| hspa4L |  |  |  |  | 100.0 | 26.0 | 27.6 | 27.5 | 28.4 | 29.6 | 26.6 | 29.4 | 27.2 | 20.3 | 18.9 | 24.8 |
| hsc70 |  |  |  |  |  | 100.0 | 42.5 | 63.4 | 35.1 | 63.2 | 93.7 | 89.7 | 48.0 | 20.5 | 18.4 | 25.5 |
| hspa13 |  |  |  |  |  |  | 100.0 | 41.9 | 29.6 | 41.8 | 42.0 | 40.2 | 35.6 | 21.1 | 20.3 | 24.9 |
| hspa5 |  |  |  |  |  |  |  | 100.0 | 33.6 | 95.1 | 63.2 | 63.5 | 49.4 | 19.8 | 19.9 | 27.6 |
| hspa14 |  |  |  |  |  |  |  |  | 100.0 | 33.4 | 35.1 | 36.1 | 31.3 | 22.5 | 18.6 | 26.8 |
| hspa5L |  |  |  |  |  |  |  |  |  | 100.0 | 63.1 | 62.3 | 48.3 | 21.5 | 20.9 | 27.5 |
| hspa8a |  |  |  |  |  |  |  |  |  |  | 100.0 | 89.7 | 51.7 | 18.9 | 19.4 | 24.3 |
| hspa8b |  |  |  |  |  |  |  |  |  |  |  | 1.00.0 | 50.4 | 20.6 | 20.2 | 24.8 |
| hspa9 |  |  |  |  |  |  |  |  |  |  |  |  | 100.0 | 22.6 | 20.5 | 23.5 |
| hspa12a |  |  |  |  |  |  |  |  |  |  |  |  |  | 1.00.0 | 31.3 | 20.1 |
| hspa12b |  |  |  |  |  |  |  |  |  |  |  |  |  |  | 100.0 | 17.3 |
| hyou1 |  |  |  |  |  |  |  |  |  |  |  |  |  |  |  | 100.0 |
