# Supplementary material for: Genome-wide identification of Hsp70/110 genes in rainbow trout and their regulated expression in response to heat stress
Source: PeerJ. 2020 Oct 23;8:e10022. doi: 10.7717/peerj.10022 (PMC7587057; doi:10.7717/peerj.10022)
Supplement: Supplemental Information 4 [file peerj-08-10022-s004.docx]

**Table S4 Protein sequence similarity of Hsp70/110 genes**

| aa% Identity | hsp70a | hsp70b | hspa4 | hspa1 | hspa4L | hsc70 | hspa13 | hspa5 | hspa14 | hspa5L | hspa8a | hspa8b | hspa9 | hspa12a | hspa12b | hyou1 |
| --- | --- | --- | --- | --- | --- | --- | --- | --- | --- | --- | --- | --- | --- | --- | --- | --- |
| hsp70a | 100.0 | 99.1 | 50.5 | 95.4 | 50.4 | 92.3 | 60.8 | 80.2 | 56.5 | 81.0 | 92.4 | 94.4 | 66.9 | 37.5 | 32.1 | 44.0 |
| hsp70b |  | 100.0 | 50.2 | 94.6 | 50.7 | 92.0 | 60.8 | 80.2 | 56.6 | 80.8 | 92.1 | 94.3 | 67.0 | 36.5 | 33.0 | 43.5 |
| hspa4 |  |  | 100.0 | 052.7 | 64.9 | 49.9 | 48.9 | 49.1 | 49.7 | 48.6 | 49.8 | 51.1 | 66.0 | 37.6 | 34.4 | 41.9 |
| hspa1 |  |  |  | 100.0 | 44.3 | 92.4 | 60.6 | 80.3 | 56.6 | 80.5 | 92.7 | 94.4 | 47.4 | 33.6 | 35.7 | 42.7 |
| hspa4L |  |  |  |  | 100.0 | 43.7 | 50.7 | 44.3 | 46.4 | 48.2 | 45.3 | 49.9 | 46.4 | 21.8 | 32.2 | 45.9 |
| hsc70 |  |  |  |  |  | 100.0 | 60.7 | 79.4 | 56.5 | 80.6 | 97.4 | 95.6 | 66.0 | 33.7 | 30.5 | 43.7 |
| hspa13 |  |  |  |  |  |  | 100.0 | 61.2 | 48.5 | 60.5 | 61.2 | 60.2 | 59.1 | 35.3 | 31.8 | 48.4 |
| hspa5 |  |  |  |  |  |  |  | 100.0 | 55.0 | 96.8 | 80.0 | 81.4 | 68.2 | 37.2 | 33.4 | 45.4 |
| hspa14 |  |  |  |  |  |  |  |  | 100.0 | 55.0 | 57.0 | 57.0 | 52.2 | 35.1 | 32.2 | 43.9 |
| hspa5L |  |  |  |  |  |  |  |  |  | 100.0 | 81.3 | 81.4 | 67.6 | 37.8 | 34.8 | 46.4 |
| hspa8a |  |  |  |  |  |  |  |  |  |  | 100.0 | 96.2 | 69.2 | 32.6 | 32.6 | 43.6 |
| hspa8b |  |  |  |  |  |  |  |  |  |  |  | 100.0 | 68.5 | 34.9 | 33.0 | 43.0 |
| hspa9 |  |  |  |  |  |  |  |  |  |  |  |  | 100.0 | 39.1 | 36.1 | 42.6 |
| hspa12a |  |  |  |  |  |  |  |  |  |  |  |  |  | 100.0 | 50.0 | 36.9 |
| hspa12b |  |  |  |  |  |  |  |  |  |  |  |  |  |  | 100.0 | 31.8 |
| hyou1 |  |  |  |  |  |  |  |  |  |  |  |  |  |  |  | 100.0 |
