# Supplementary material for: Genome-wide identification of Hsp70/110 genes in rainbow trout and their regulated expression in response to heat stress
Source: PeerJ. 2020 Oct 23;8:e10022. doi: 10.7717/peerj.10022 (PMC7587057; doi:10.7717/peerj.10022)
Supplement: Supplemental Information 5 [file peerj-08-10022-s005.doc]

**Table S5** RNA-Seq Expression Data

| Gene | Gene id | CL1 | CL2 | CL3 | HL1 | HL2 | HL3 | CHK1 | CHK2 | CHK3 | HHK1 | HHK2 | HHK3 |
| --- | --- | --- | --- | --- | --- | --- | --- | --- | --- | --- | --- | --- | --- |
| hsp70a | NM_001124228.1 | 8.529647 | 5.823915 | 12.437546 | 50.762125 | 20.906671 | 43.360942 | 73.310418 | 54.775653 | 82.7171976 | 414.93733 | 326.733600 | 304.736511 |
| hspa4 | XM_021559693.1 | 40.90232 | 35.23468 | 46.423391 | 118.63341 | 92.434975 | 57.763576 | 25.62631 | 37.944083 | 33.387344 | 234.85661 | 185.374029 | 174.208617 |
| hspa4L | XM_021574292.1 | 2.926089 | 3.656269 | 2.8091305 | 8.7027129 | 5.6421223 | 9.2463692 | 27.431456 | 38.930273 | 28.236212 | 116.65771 | 99.020969 | 93.4033924 |
| hspa5 | XM_021590926.1 | 12.57828 | 30.95352 | 20.187978 | 129.24570 | 81.71062 | 341.5339 | 33.049070 | 32.676260 | 32.390504 | 145.23304 | 88.168616 | 53.3992441 |
| hspa8a | XM_021617785.1 | 14.06415 | 11.381081 | 15.809729 | 82.005657 | 32.548681 | 62.73674 | 113.09431 | 103.581710 | 141.02542 | 646.767274 | 490.5060623 | 434.159760 |
| hspa8b | XM_021624823.1 | 7.575673 | 20.23044 | 5.438981 | 232.016936 | 62.388482 | 51.952584 | 62.831776 | 27.341042 | 41.978177 | 93.4535600 | 173.612146 | 125.564427 |
| hspa9 | XM_021560806.1 | — | — | — | — | — | — | 15.110151 | 60.683542 | 34.855909 | 354.379090 | 413.702964 | 189.400512 |

Note: Read abundance is showed in terms of expected number of fragments per kilobase of transcript sequence per millions base pairs sequence (FPKM)
